# How are your berries? Perspectives of Alaska's environmental managers on trends in wild berry abundance

**DOI:** 10.3402/ijch.v74.28704

**Published:** 2015-09-15

**Authors:** Jerry Hupp, Michael Brubaker, Kira Wilkinson, Jennifer Williamson

**Affiliations:** 1 U.S. Geological Survey, Alaska Science Center, Anchorage, AK, USA; 2 Alaska Native Tribal Health Consortium, Anchorage, AK, USA; 3 Alaska Native Science and Engineering Program, University of Alaska Anchorage, Anchorage, AK, USA

**Keywords:** wild berries, Alaska, climate change, environmental survey

## Abstract

**Background:**

Wild berries are a valued traditional food in Alaska. Phytochemicals in wild berries may contribute to the prevention of vascular disease, cancer and cognitive decline, making berry consumption important to community health in rural areas. Little was known regarding which species of berries were important to Alaskan communities, the number of species typically picked in communities and whether recent environmental change has affected berry abundance or quality.

**Objective:**

To identify species of wild berries that were consumed by people in different ecological regions of Alaska and to determine if perceived berry abundance was changing for some species or in some regions.

**Design:**

We asked tribal environmental managers throughout Alaska for their views on which among 12 types of wild berries were important to their communities and whether berry harvests over the past decade were different than in previous years. We received responses from 96 individuals in 73 communities.

**Results:**

Berries that were considered very important to communities differed among ecological regions of Alaska. Low-bush blueberry (*Vaccinium uliginosum* and *V. caespitosum*), cloudberry (*Rubus chamaemorus*) and salmonberry (*Rubus spectabilis*) were most frequently identified as very important berries for communities in the boreal, polar and maritime ecoregions, respectively. For 7 of the 12 berries on the survey, a majority of respondents indicated that in the past decade abundance had either declined or become more variable.

**Conclusions:**

Our study is an example of how environmental managers and participants in local observer networks can report on the status of wild resources in rural Alaska. Their observations suggest that there have been changes in the productivity of some wild berries in the past decade, resulting in greater uncertainty among communities regarding the security of berry harvests. Monitoring and experimental studies are needed to determine how environmental change may affect berry abundance.

Climate warming has been amplified in northern latitudes, including Alaska (1,2) and may result in ecological changes that will profoundly affect the wild foods that are important to rural communities (3,4). Understanding how subsistence resources have been affected by recent climate change, and may be affected by changes yet to come (5), is necessary to plan for food security in Alaska.

Evaluating changes to subsistence resources across a region as large and ecologically diverse as Alaska is challenging. However throughout Alaska, government, tribal and private environmental managers live and work in rural communities. These individuals are knowledgeable about the local environment and can provide a network for reporting on environmental change. Here, we report the observations of Alaska's environmental managers on recent trends in wild berry abundance.

Wild berries are valued as a traditional food and for medicinal purposes in rural Alaska, and berry picking is an important cultural activity (6–8). Berries add diversity to the diets of people in rural areas and are an important alternative to domestic fruits, which in remote areas can be difficult to obtain and are expensive. Many species of wild berries have high antioxidant activity (9–12) that has been shown to reduce the risk of diabetes, heart disease, cognitive decline and cancer (13–15). Consumption of wild berries has been encouraged as a means to improve health in Alaska's rural communities (16). Berries also provide food to wildlife species that are important to subsistence harvest (17–19). Thus, wild berries are important to cultural heritage, food security and human health in rural Alaska.

Despite their importance to rural communities, we know little regarding how berry-producing plants may be affected by climate change. Change in precipitation and temperature could affect berry production (20–22), as could changes in pollination rates (23). Change in berry harvests could serve as an indicator of environmental modification at a landscape level.

In Alaska, there are approximately 50 species of plants that produce berries (24). People regularly consume berries from about half of those species (25). However, there is little published information regarding which species of berries are important to communities in different regions of the state. That information was needed to guide further monitoring and research on the effects of climate change on berry resources. Therefore, we conducted a survey in which we asked local environmental managers throughout Alaska: (a) to identify which species of berries were important to people in their community and (b) whether they perceived that the abundance of berries had changed in recent years. Our goals were to contrast species composition of the berry harvest among ecological regions of Alaska, and identify regions or species for which berry abundance was perceived to be changing.

## Methods

### Survey approach

Between November 2013 and February 2014, we distributed a survey to 3 groups of environmental managers that were knowledgeable about berries and their use: (a) the Local Environmental Observer (LEO) Network of the Alaska Native Tribal Health Consortium, (b) attendees of the Alaska Tribal Conference on Environmental Management (ATCEM) and (c) attendees of the Alaska Forum on the Environment (AFE). People in these groups were often tribal environmental professionals affiliated with the Environmental Protection Agency's Indian General Assistance Program. The survey was provided to the LEO network in an online format, whereas paper copies were distributed upon request to interested individuals at ATCEM and AFE. Contents of the online and paper surveys were the same. In conjunction with distribution of the survey, we held either an online webinar (LEO) or classroom seminars (ATCEM and AFE) to explain the species covered by the survey and its intent.

In the survey, we first asked people to identify the community in which they lived and the number of years they had lived there. We then asked questions about 12 types of berries that we believed were most likely to be picked in various regions of Alaska (see Supplementary file). Some berries were combinations of species that were closely related and similar in appearance. We selected berries for inclusion in the survey based on discussions with berry users, on Pratt's (25) description of the palatability and use of berries, and from our personal knowledge. For each type of berry, we provided a picture of its fruit, a note on height of the plant, a map of its known distribution based on Hultén (24) and frequently used English common names. We asked if the berry occurred near the respondent's community. If they responded positively, we asked that they classify the importance of the berry to their community as either:

- It is very important. It is the berry that people pick most often
- It is important. People sometimes pick this berry.
- It is not important. People rarely pick this berry.

If the respondent indicated that people in their community picked a berry, we asked that they provide their perception of whether abundance and harvests of that berry had changed over the past 10 years:

- It is more abundant. The berry harvest is larger now than in previous years.
- It is less abundant. We pick fewer berries of this species than we used to.
- Its abundance has not changed.
- Its abundance is more variable from 1 year to the next than it used to be.

We considered that increased abundance or no change in abundance were indicators of certainty in berry harvests, and suggested that a family could plan whether to incorporate that berry into their diet. Conversely, decreased abundance or increased variability in abundance indicated uncertainty in harvests, and suggested that a family could not plan on that berry being available. For each berry, we also asked participants to indicate the volume of berries that families in their community picked on average, and the distance people travelled to the best berry-picking areas.

### Data analysis

**Fig. 1:**
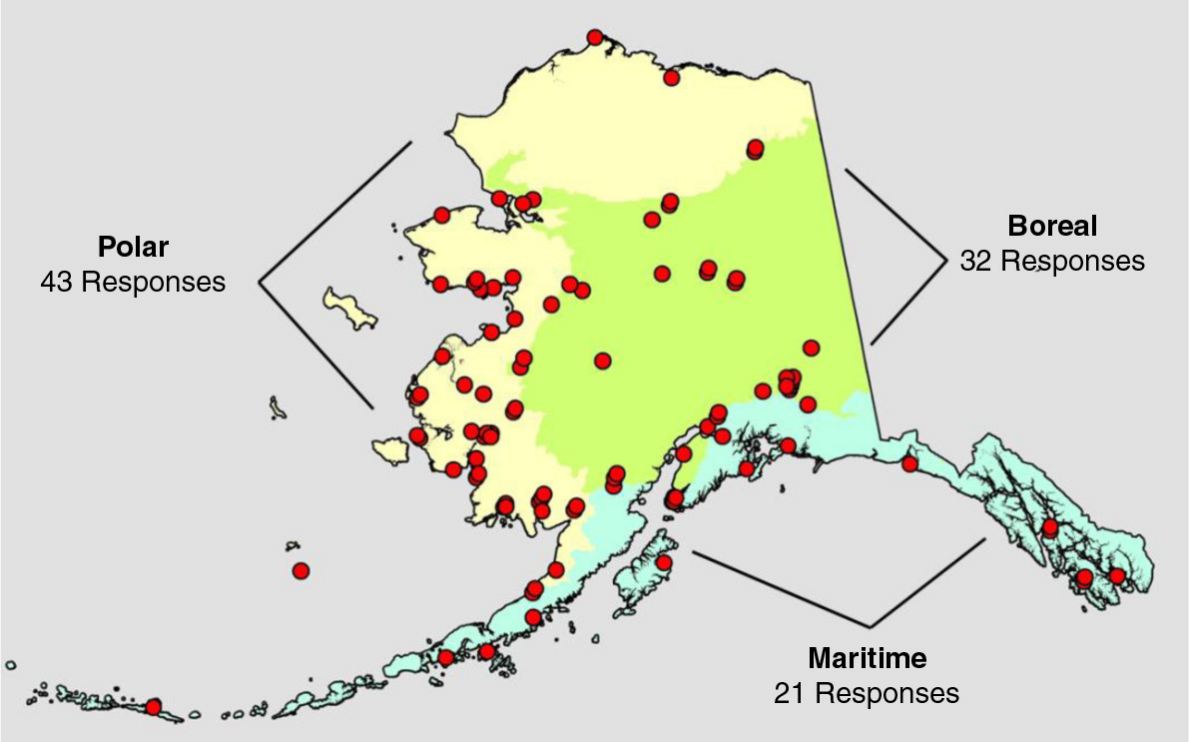
Alaskan communities where environmental observers completed surveys on berry importance and abundance. Multiple responses from a single community are offset slightly to indicate where they occurred. The number of responses from each ecoregion of Alaska is indicated.

We summarized regional differences in berry importance based on Level I ecoregions of Alaska (26) (Fig. 1). This classification broadly divides Alaska into 3 ecological zones (26) that also reflect distributions of Native cultures (27) and some berry species (24). The polar ecoregion of northern and western Alaska is a treeless, windswept region typically underlain by permafrost and in which land cover is mainly sedge and grass tundra, low shrubs and wetlands. It includes the montane regions of the Brooks Range. Native people who live in the polar ecoregion mainly include the Inupiaq of northern Alaska and the Yup'ik of Cup'ik people of western Alaska. The boreal ecoregion of interior Alaska is dominated by spruce and birch forests, but it includes alpine tundra in mountainous regions. There are many areas in the boreal ecoregion where forests give way to sedge meadows and ericaceous shrubs in poorly drained sites. Numerous groups of Athabascan people live in the boreal ecoregion. The maritime ecoregion occurs along the southern coast of Alaska and includes the more windswept areas of the maritime ecoregion on the Alaska Peninsula and Aleutian Islands of southwest Alaska. Tlingit, Haida and Tsimshian mainly live in southeast Alaska. The Unangax and Alutiiq people mainly live in the Alaska Peninsula, Kodiak Archipelago and Aleutian Islands of southwest Alaska.

For each berry on the survey, we computed the percentage of respondents within an ecoregion who considered the berry as very important to their communities. Within each ecoregion, we identified berries that were considered to be very important by >50% of respondents. For those berries, we computed the percentage of ecoregional responses in each category for change in abundance, gallons harvested and distance travelled. For all berries on the survey, we computed the percentage of statewide responses in each category of change in abundance.

## Results

### Identification of very important berries

**Fig. 2:**
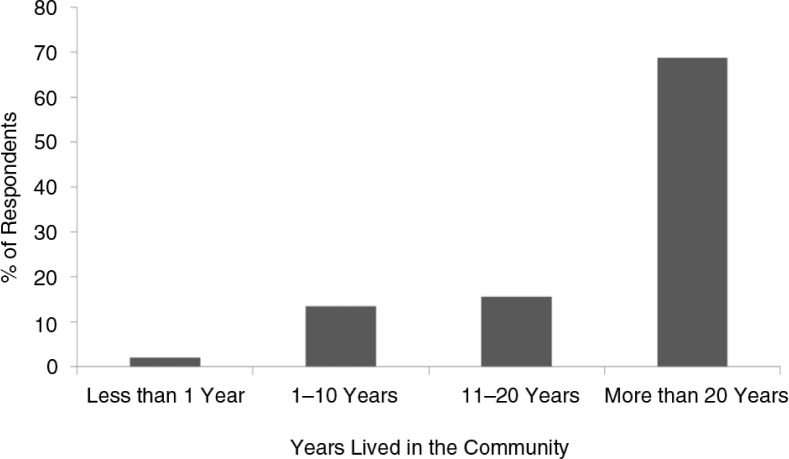
Number of years that respondents to the berry survey had lived in their communities.

We received a total of 96 completed surveys from environmental managers in 73 communities (21% of the communities in Alaska). Most (69%) of the people that responded to the survey had lived in their community for >20 years (Fig. 2).

**Fig. 3:**
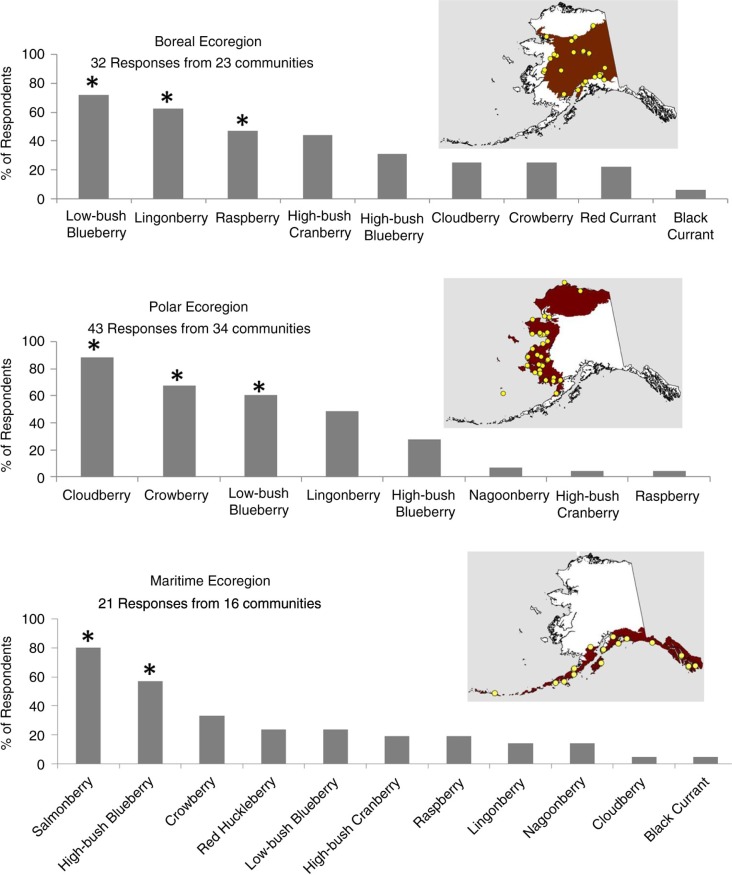
The wild berry species that are very important to communities differ among the 3 ecological regions of Alaska. Vertical bars represent the percentage of survey respondents in each ecoregion that indicated a berry was very important to their community. Inset maps show boundaries of each ecoregion and the locations of communities that responded to the survey. Asterisks indicate berries identified as very important by >50% of respondents in an ecoregion.

Each person who responded to the survey identified from 0 to 8 berries that they considered as very important to their community (median=3 berries). The berries that were very important to communities varied among ecological regions (Fig. 3). In the boreal ecoregion, low-bush blueberry (*Vaccinium uliginosum* and *V. caespitosum*), lingonberry (*V. vitis-idaea*) and raspberry (*Rubus idaeus*) were considered very important by >50% of respondents. In the polar ecoregion of western and northern Alaska, cloudberry (*Rubus chamaemorus*), crowberry (*Empetrum nigrum*) and low-bush blueberry were considered by the majority of respondents to be very important to their communities. In the maritime ecoregion, salmonberry (*R. spectabilis*) and high-bush blueberry (*V. ovalifolium* and *V. alaskensis*) were considered very important by most respondents.

### Changes in berry abundance

**Fig. 4:**
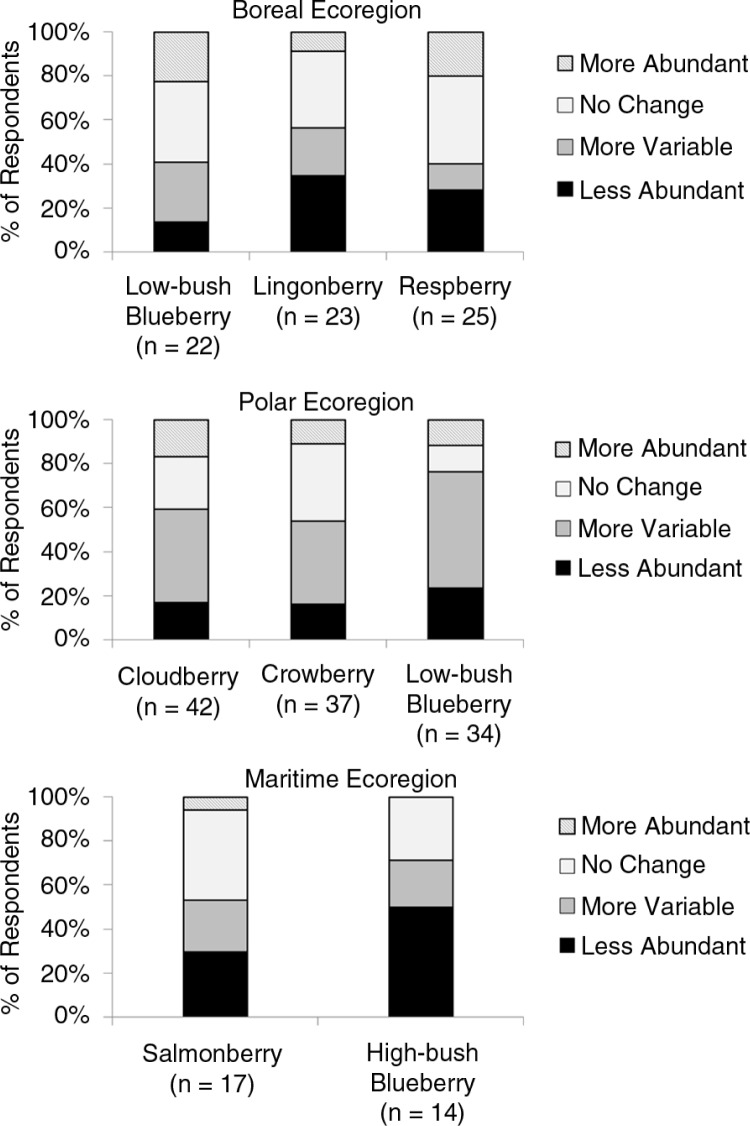
Perceived change in abundance for berries that were identified as very important by >50% of respondents in an Alaskan ecoregion. Percentages are based on responses received for that berry within an ecoregion. The number of people that answered questions on change in abundance in each ecoregion is indicated beneath each berry.

There was little consensus regarding change in abundance for the important berries in each ecoregion (Fig. 4). No change in abundance was the most common perception for the berries most frequently identified as very important in the boreal ecoregion, whereas increased variability was the most common perception for the very important berries in the polar ecoregion. In the maritime ecoregion, people most commonly perceived that abundance of salmonberry had not changed, whereas abundance of high-bush blueberry had declined.

**Fig. 5:**
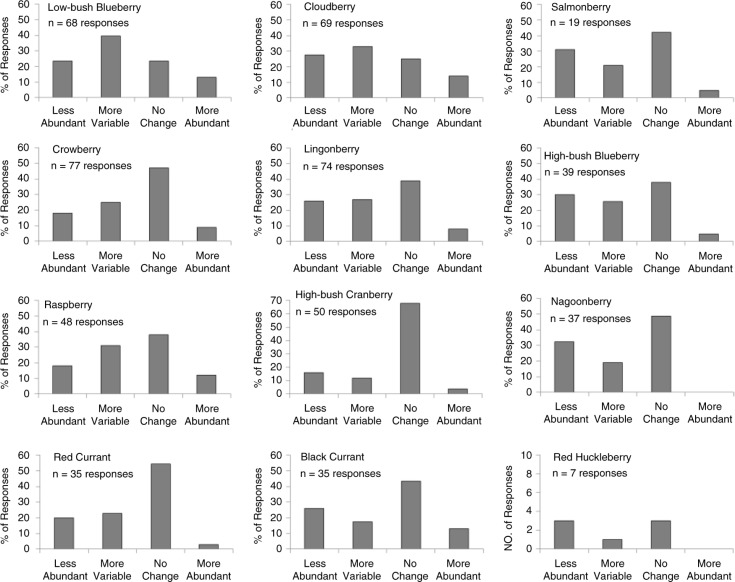
Perceived change in abundance for all berries included in the survey, based on the statewide response for that berry. Vertical bars are the percentage of respondents that indicated a change in berry abundance given that the berry occurred near their community. Percentages are not indicated for red huckleberry, due to the small number of respondents for that berry. The number of statewide responses for each berry is indicated.

Although there was no consensus on trends in abundance, many people responded in a manner that suggested berry harvests had become less certain in the past decade, either due to declining abundance or increased annual variation in abundance. For lingonberry in the boreal ecoregion and for each of the berries that were most frequently identified as very important in the polar and maritime regions, the majority of respondents in those regions (53–76%) perceived that berry abundance had either declined or become more variable (Fig. 4). On a statewide basis, 67% of respondents indicated that at least one berry that was very important to their community had either declined in abundance or that abundance had become more variable in the past decade. For 7 of the 12 berries on our survey, the number of people statewide who perceived that abundance had declined or become increasingly variable was greater than the number that indicated abundance had increased or not changed (Fig. 5).

### Volume of berries picked and distance to berry-picking areas

**Fig. 6:**
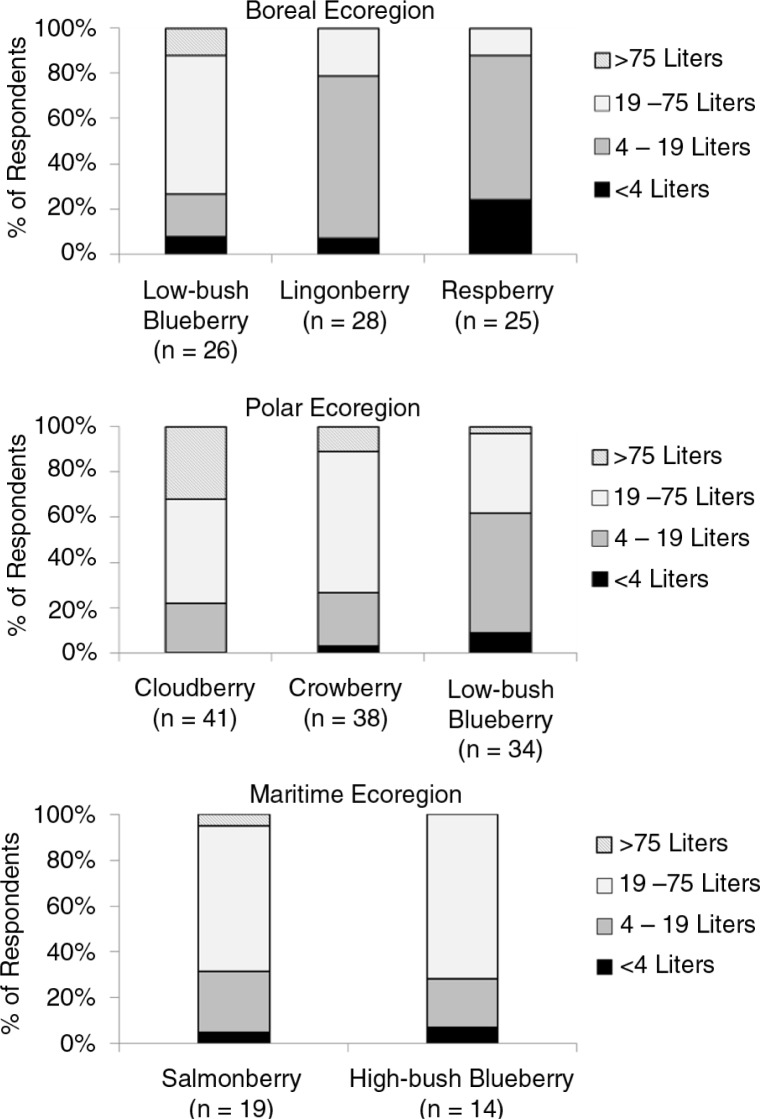
Volume of berries collected by families in each ecoregion of Alaska. Only berries indicated as very important by >50% of respondents in an ecoregion are shown. The number of people in each ecoregion that answered questions on volume of berries is indicated beneath each species.

**Fig. 7:**
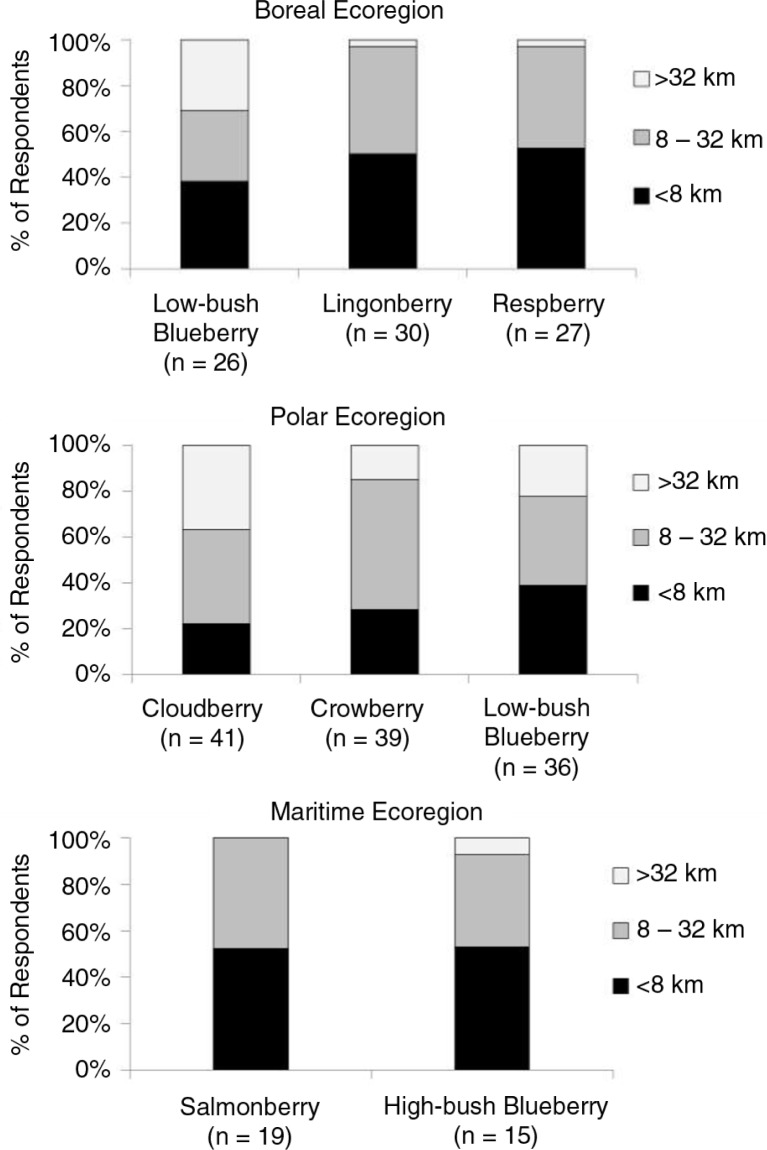
Distance between a community and the best places to pick berries in each ecoregion of Alaska. Only berries indicated as very important by >50% of respondents in an ecoregion are shown. The number of people in each ecoregion that answered questions on distance to berry-picking sites is indicated beneath each species.

For the most important berry in each ecoregion, the majority of families picked at least 19 litres of berries (Fig. 6). However, some families picked >75 litres, especially for cloudberry in the polar ecoregion. In each region, the majority of people indicated that the best berry-picking sites for each of the important berries were within 32 km of their community (Fig. 7).

## Discussion

The people who participated in our survey were mainly environmental professionals who had long associations with their communities. Although participants were not randomly selected from berry users in Alaska, thus precluding statistical inference to a larger population, they were individuals that were highly knowledgeable about local resources and harvests. Their insights are useful given that there is little published information on species composition or changes in abundance of wild berries that are harvested in rural Alaska.

People from across Alaska responded to the survey and most identified >1 type of berry that was very important to his or her community. The majority of respondents indicated that families in their communities picked ≥19 litres of one or more species of berry. These results illustrate not only the broad geographic importance of berries to the state's rural communities, but also indicate that berries can significantly contribute to a families’ diet. The latter is especially true given that berries are often extended by mixing them with other foods to make agutak (Eskimo ice cream), jams and pies. People typically picked berries within 32 km of their community. Compared to other subsistence foods that people often have to travel longer distances to obtain, berries are important as a locally available resource that can be harvested with lower fuel costs (28).

No single berry dominated harvests throughout Alaska. In each ecological region, and in some cases at the community level, there was a unique mix of favoured berries. Differences in the composition of berry harvest among ecological regions likely reflect differences in abundance of berry species. Cloudberry, crowberry and low-bush blueberry, the favoured species in the polar ecoregion, are distributed throughout Alaska. However, they are typically more abundant in the bog, tundra, heath and low-shrub communities that mainly comprise the polar ecoregion (24,25,29). The distributions of salmonberry and high-bush blueberry, the most important species in the maritime ecoregion, are restricted to the temperate and subalpine forests, and coastal communities of southern Alaska (24,30). In the maritime ecoregion, crowberry was also important to communities on the Alaska Peninsula and Aleutian Islands where this species is common (19,31). Low-bush blueberry, lingonberry and raspberry, the favoured species in the boreal ecoregion, are common bog and forest understory plants (32). Low-bush blueberry and lingonberry also occur in alpine tundra of the boreal ecoregion. Although local abundance is likely an important determinant of the species composition of berry harvests, cultural preferences could also influence berry selection.

Because environmental variables that influence berry production may vary among species (21), different species of berries may respond differently to climate warming. Our identification of important berries in each ecoregion is useful to select species that should be the focus of further monitoring and experimental work aimed towards predicting how berry resources will be affected by climate change.

There was no agreement among observers within an ecoregion on whether abundance of a berry had changed. Even within communities, different observers often perceived changes differently for the same berry. This may partly reflect individual experiences with changes in berry harvests. Berry abundance can be highly variable across small geographic areas, possibly due to site-specific differences in environmental conditions (19). Localized changes in productivity of individual berry patches may cause different observers to reach disparate conclusions on whether berry abundance has changed over time.

Although there was no agreement on trends in berry harvests, there was little to suggest that abundance had improved for any berry in recent years. Increased abundance was typically the least common response for each berry. Rather, for 7 of the 12 berries in our survey, the most common perception statewide was that berry harvests had become less reliable either due to declining abundance or increased annual variability. Flint et al. (8) and Parlee et al. (33) reported that people in some Alaskan and Canadian communities were concerned about the effects of climate warming on berry harvests. Our findings suggest that recent changes in the abundance of some berries may provide a basis for those concerns. That perceived trends were dissimilar among berries is not surprising given that interspecific differences in reproductive strategies could influence how berry production is influenced by environmental change. For example, insect-pollinated species such as low-bush blueberry or cloudberry (23,34) may respond differently to environmental variation than species that are mainly wind pollinated such as crowberry (29), especially if weather conditions affect insect pollinators (35).

There was some evidence of regional differences in berry trends. The perception that abundance had declined or become increasingly variable was more common for important berries in the polar and maritime ecoregions than in the boreal ecoregion. Low-bush blueberry was considered very important by >50% of respondents in both the boreal and polar ecoregions. However, the percentage of respondents who perceived abundance of low-bush blueberry to be declining or becoming increasingly variable was almost twice as large in the polar ecoregion (76%) than in the boreal ecoregion (41%). Thus, there may be underlying ecological changes that are having greater effects on berry resources in the coastal and tundra regions of Alaska. This is plausible given the large-scale environmental changes associated with temperature increase, permafrost deterioration, and coastal flooding and erosion in those regions (36–38). Long-term declines in cloudberry and some other northern berry species have also been reported in Finland (20).

The survey neither does allow us to quantify the magnitude of change in berry abundance, nor clearly identify specific environmental conditions that may have affected berry productivity. Spatial and temporal patterns in production of many berries are inherently variable (19–21). Increased variability in berry harvests does not necessarily mean that average harvests have declined. But, it does indicate that planning on a harvest is more difficult. Weather patterns in Alaska are expected to become more variable as a result of climate warming (39,40). The increase in annual variation in berry abundance reported by many people may be evidence that on-going climatic fluctuation has influenced recent harvests of some berries. Increased amplitude in climatic fluctuation may exacerbate variation, resulting in greater uncertainty regarding future berry harvests.

## Conclusions

Our survey highlights the importance of wild berries to rural communities throughout Alaska, and demonstrates the value of local environmental managers in monitoring resource change. In many communities, people perceive that over the past decade berry harvests have declined or have become more variable. As a result, people may be increasingly uncertain about future berry harvests. To better understand how berry harvests may be affected by environmental change, we recommend that (a) annual surveys of environmental managers and other berry users be conducted to monitor harvest trends (b), site-specific measures of plant species composition and berry abundance be conducted to correlate berry harvest with environmental variables and to detect plant community change and (c) experimental manipulations be increasingly conducted to identify environmental drivers of berry productivity and to develop predictive models of the effects of climate change on berry resources. We recommend that future monitoring and experimental studies focus on species that were identified as very important to communities in our survey. Also, that methods by which rural communities can increase their resilience to declining or more variable berry harvests be explored.

## Supplementary Material

How are your berries? Perspectives of Alaska's environmental managers on trends in wild berry abundance

## References

[1] Committee on Ecological Impacts of Climate Change (2008). Ecological impacts of climate change.

[2] Bieniek PA, Walsh JE, Thoman RL, Bhatt US (2014). Using climate divisions to analyze variations and trends in Alaska temperature and precipitation. J Clim.

[3] Hinzman LD, Bettez ND, Bolton WR, Chapin FS, Dyurgerov MB, Fastie CL (2005). Evidence and implications of recent climate change in northern Alaska and other arctic regions. Clim Change.

[4] Kofinas GP, Chapin FC, BurnSilver S, Schmidt JI, Fresco NL, Kielland K (2010). Resilience of Athabascan subsistence systems to interior Alaska's changing climate. Can J Forest Res.

[5] Intergovernmental Panel on Climate Change (2014). Climate change 2014: impacts, adaptation, and vulnerability. Part A: Global and sectoral aspects. Contribution of Working Group II to the Fifth Assessment Report of the Intergovernmental Panel on Climate Change.

[6] Thornton TF (1999). Tleikwaaní, the “berried” landscape: the structure of Tlingit edible fruit resources at Glacier Bay, Alaska. J Ethnobiol.

[7] Redwood DG, Ferucci ED, Schumacher MC, Johnson JS, Lanier AP, Helzer LJ (2008). Traditional foods and physical activity patterns and associations with cultural factors in a diverse Alaska Native population. Int J Circumpolar Health.

[8] Flint CG, Robinson ES, Kellogg J, Ferguson G, BouFajreldin L, Dolan M (2011). Promoting wellness in Alaskan villages: integrating traditional knowledge and science of wild berries. EcoHealth.

[9] Leiner RH, Holloway RS, Neal DB (2008). Antioxident capacity and quercetin levels in Alaska's wild berries. Int J Fruit Sci.

[10] Ogawa K, Sakakibara H, Iwata R, Ishii T, Sato T, Goda T (2008). Anthocyanin composition and antioxidant activity of the crowberry (*Empetrum nigrum*) and other berries. J Agr Food Chem.

[11] Kellogg J, Wang J, Flint C, Ribnicky D, Kuhn P, Gonzalez De Mejia E (2010). Alaskan wild berry resources under the cloud of climate change. J Agr Food Chem.

[12] Dinstel RR, Cascio J, Koukel S (2013). The antioxidant level of Alaska's wild berries: high, higher, and highest. Int J Circumpolar Health.

[13] Hertog MG, Kromhout D, Aravanis C, Blackburn H, Buzina R, Fidanza F (1995). Flavonoid intake and long-term risk of coronary heart disease and cancer in the seven countries study. Arch Internal Med.

[14] Neto CC (2007). Cranberry and blueberry: evidence for protective effects against cancer and vascular diseases. Mol Nutr Food Res.

[15] Devore DD, Kang JH, Bretleler MMB, Grodstein F (2012). Dietary intakes of berries and flavonoids in relation to cognitive decline. Ann Neurol.

[16] Johnson JS, Nobmann ED, Asay E, Lanier AP (2009). Dietary intake of Alaska Native people in two regions and implications for health: the Alaska Native dietary and subsistence food assessment project. Int J Circumpolar Health.

[17] Nelson UC, Hansen HA (1959). The cackling goose – its migration and management. Trans N Am Wildl Nat Res Conf.

[18] Weeden RB (1969). Foods of rock and willow ptarmigan in central Alaska with comments on interspecific competition. Auk.

[19] Hupp JW, Safine DE, Nielson RM (2013). Response of cackling geese (*Branta hutchinsii taverneri*) to spatial and temporal variation in the production of crowberries on the Alaska Peninsula. Polar Biol.

[20] Wallenius TH (1999). Yield variations of some common wild berries in Finland in 1956–1996. Ann Bot Fenn.

[21] Krebs CR, Boonstra R, Cowcill K, Kenney AJ (2009). Climatic determinants of berry crops in the boreal forest of southwestern Yukon. Botany.

[22] Holden ZA, Kasworm WF, Servheen C, Hahn B, Dobrowski S (2012). Sensistivity of berry productivity to climatic variation in the Cabinet-Yaak grizzly bear recovery zone, northwest United States, 1989–2010. Wildl Soc Bull.

[23] Brown AO, McNeil JN (2009). Pollination ecology the high latitude dioecious cloudberry (*Rubus chamaemorus*; Rosacea). Am J Bot.

[24] Hultén E (1968). Flora of Alaska and neighboring territories.

[25] Pratt V (1995). Alaska's wild berries and berry-like fruit.

[26] Nowacki G, Spencer P, Fleming M, Brock T, Jorgenson T (2001). Ecoregions of Alaska. U.S. Geological Survey Open-File Report 02-297 (map).

[27] Krauss ME (1982). Native peoples and languages of Alaska. Alaska Native Language Center.

[28] Brinkman T, Maracle KB, Kelly J, Vandyke M, Firmin A, Springsteen A (2014). Impact of fuel costs on high-latitude subsistence activities. Ecol Soc.

[29] Bell JNB, Tallis JH (1973). Biological flora of the British Isles: *Empetrum nigrum*. J Ecol.

[30] Vander Kloet SP, Dickinson TA (1999). The taxonomy of *Vaccinium* section Myrtillus (Ericaceae). Brittonia.

[31] Talbot S, Talbot SL (1994). Numerical classification of the coastal vegetation of Attu Island, Aleutian Islands, Alaska. J Veg Sci.

[32] Chapin FS, Hollingsworth T, Murray DF, Viereck LA, Walker MD, Chapin FS, Oswood MW, Van Cleve K, Viereck LA, Verbyla DL (2006). Floristic diversity and vegetation distribution in the Alaskan boreal forest. Alaska's changing boreal forest.

[33] Parlee B, Berkes F, Teetl'it Gwich'in Renewable Resources Council (2005). Health of the land, health of the people: a case study on Gwich'in berry harvesting in Northern Canada. EcoHealth.

[34] Jacquemart A (1997). Pollen limitation in three sympatric species of *Vaccinium* (Ericaceae) in the Upper Ardennes, Belgium. Plant Syst Evol.

[35] Totlan Ø (1994). Influence of climate, time of day and season, and flower density on insect flower visitation in alpine Norway. Arctic Alpine Res.

[36] Lawrence DM, Slater AG, Tomas RA, Holland MM, Deser C (2008). Accelerated Arctic land warming and permafrost degradation during rapid sea ice loss. Geophys Res Lett.

[37] Jones BM, Arp CD, Jorgenson MT, Hinkel KM, Schmutz JA, Flint PL (2009). Increase in the rate and uniformity of coastline erosion in Arctic Alaska. Geophys Res Lett.

[38] Terenzi J, Jorgenson MT, Ely CR (2014). Storm-surge flooding on the Yukon-Kuskokwim Delta, Alaska. Arctic.

[39] Porter DF, Cassano JJ, Serreze MC (2012). Local and large-scale atmospheric responses to reduced Arctic sea ice and ocean warming in the WRF model. J Geophys Res.

[40] Chapin FS, Trainor SF, Cochran P, Huntington H, Markon C, McCammon M, Melillo JM, Richmond TC, Yohe GW Chapter 22: Alaska. Climate change impacts in the United States: The Third National Climate Assessment.

