# Supplementary material for: How are your berries? Perspectives of Alaska's environmental managers on trends in wild berry abundance
Source: Int J Circumpolar Health. 2015 Sep 15;74:10.3402/ijch.v74.28704. doi: 10.3402/ijch.v74.28704 (PMC4574151; doi:10.3402/ijch.v74.28704)
Supplement: How are your berries? Perspectives of Alaska's environmental managers on trends in wild berry abundance [file IJCH-74-28704-s001.doc]

**Supplemental Information**

We analyzed results based on the following 12 berries. Some berries that were similar in appearance included >1 species.

1. Low-bush blueberry (*Vaccinium uliginosum* and V*. caespitosum*)

2. Cloudberry (*Rubus chamaemorus*)

3. Crowberry (*Empetrum nigrum*)

4. Lingonberry (*Vaccinium vitis-idaea*)

5. High-bush blueberry (*Vaccinium ovalifolium and V. alaskensis*)

6. Raspberry (*Rubus idaeus*)

7. High-bush cranberry (*Viburnum edule*)

8. Salmonberry (*Rubus spectabilis*)

9. Red currant (*Ribes triste*)

10. Nagoonberry (*Rubus arcticus*)

11. Red Huckleberry (*Vaccinium parvifoliu*m)

12. Black currant (*Ribes laxiflorum*, *R. hudsonianum*, and *R. lacustre*).

In the survey, we separately presented questions about early blueberry (*V. ovalifolium*) and Alaska blueberry (*V. alaskensis*). We likewise separately presented questions about trailing black currant (*R. laxiflorum*), northern black currant (*R. hudsonianum*), and swamp gooseberry (*R. lacustre*). Upon receipt of completed surveys we found some people indicated they could not distinguish between species within these groups. Also, when an individual responded to >1 species in a group, responses were typically similar between species. Therefore, we combined responses for early blueberry and Alaska blueberry into “high-bush blueberry”. Currants and gooseberry were pooled into “black currant”. If a person completed responses for only one species in these groups, we used those responses to represent the group for our analysis. If a person provided answers for >1 species in a group, and responses differed, we used the responses associated with the berry that was identified as the more important in the group.
